# The Human Genome Organisation (HUGO) and a vision for Ecogenomics: the Ecological Genome Project

**DOI:** 10.1186/s40246-023-00560-x

**Published:** 2023-12-18

**Authors:** Benjamin Capps, Ruth Chadwick, Zohar Lederman, Tamra Lysaght, Catherine Mills, John J. Mulvihill, William S. Oetting, Ingrid Winship, HUGO Committee on Ethics, Law and Society

**Affiliations:** 1 Department of Bioethics, Dalhousie University, 5849 University Avenue, CRC Building, Room C-312, PO Box 15000, Halifax, NS B3H 4R2 Canada; 2 Cardiff University, Cardiff, UK; 3 University of Hong Kong, Pokfulam, Hong Kong; 4 National University of Singapore, Singapore, Singapore; 5 Monash University, Melbourne, Australia; 6 University of Oklahoma Health Sciences Center, Oklahoma City, USA; 7 University of Minnesota, Minneapolis, USA; 8 University of Melbourne, Melbourne, Australia

**Keywords:** Human Genome Organisation, Ecogenomics, Human Genome Project, Determinants of health, One Health

## Abstract

**Background:**

The following outlines ethical reasons for widening the Human Genome Organisation’s (HUGO) mandate to include ecological genomics.

**Main:**

The environment influences an organism’s genome through ambient factors in the biosphere (e.g. climate and UV radiation), as well as the agents it comes into contact with, i.e. the epigenetic and mutagenic effects of inanimate chemicals and pollution, and pathogenic organisms. Emerging scientific consensus is that social determinants of health, environmental conditions and genetic factors work together to influence the risk of many complex illnesses. That paradigm can also explain the environmental and ecological determinants of health as factors that underlie the (un)healthy ecosystems on which communities rely. We suggest that *The Ecological Genome Project* is an aspirational opportunity to explore connections between the human genome and nature. We propose consolidating a view of Ecogenomics to provide a blueprint to respond to the environmental challenges that societies face. This can only be achieved by interdisciplinary engagement between genomics and the broad field of ecology and related practice of conservation. In this respect, the One Health approach is a model for environmental orientated work. The idea of Ecogenomics—a term that has been used to relate to a scientific field of ecological genomics—becomes the conceptual study of genomes within the social and natural environment.

**Conclusion:**

The HUGO Committee on Ethics, Law and Society (CELS) recommends that an interdisciplinary One Health approach should be adopted in genomic sciences to promote ethical environmentalism. This perspective has been reviewed and endorsed by the HUGO CELS and the HUGO Executive Board.

## Background

In December 2022, the *Conference of the Parties (COP15) to the United Nations Convention on Biological Diversity* (CBD) (1993) adopted the *Kunming-Montreal Global Biodiversity Framework*.^1^ Signed in Montreal, the Framework has 23 global targets that must be achieved by 2030. These include protecting 30 per cent of terrestrial, inland water, coastal and marine areas; effectively reducing anthropogenic pollution; and minimising the impact of climate change. One of the overarching goals is to operationalise monetary and non-monetary benefits from the utilisation of genetic resources (to be ‘shared fairly and equitably’). The relevance of genetics to the environment might not be immediately obvious, but Montreal was also the forum for the *Fourth Meeting of the Parties to the Nagoya Protocol on Access to Genetic Resources and the Fair and Equitable Sharing of Benefits Arising from their Utilization* [1]. The Nagoya Protocol—also part of the CBD—is a reference point for developing global genomic research that contributes to the conservation and sustainable use of biological diversity (Article 8(a)), bringing into focus present or imminent emergencies that threaten or damage human, animal, or plant health, such as zoonotic pandemics (8(b)) and the importance of genetic resources in agriculture (8(c)).^2^ We [CELS] see COP15 as an overall turn to environmentalism that is significant for genomic sciences.

## Defining Ecogenomics

Ecogenomics, as a scientific field, is not new, although it has become a nebulous concept [2]. George Brewer defined Eco*genetics* as a necessary response to the environmental concerns such as pollution, and called for multidisciplinary research combining genetics, biochemistry, microbiology, and pharmacology [3]. *It was a novel view of ‘human ecology’.* In subsequent years, concepts of the environment have been central to fields such as ecosystem genetics and environmental DNA studies [4], and important to the development of epigenetics, epidemiology, exposomics, pharmacogenomics, and toxicogenomics [5, 6]. Genomics has also been part of the ecological conservation field [7].

COP15, however, is an opportunity to further develop a vision for genomics; and, as such, we propose not only the consolidation in the field of Ecogenomics, but an expansion of the field into social ecology and social conservation. Whereas ecology and conservation were once related to non-human fields, they are now orientated to the connections between the social lives of human beings and non-human animals and the environments they share [8]. These fields have become central to the idea of One Health:

‘One Health is an integrated, unifying approach that aims to sustainably balance and optimize the health of people, animals, and ecosystems. It recognizes the health of humans, domestic and wild animals, plants, and the wider environment (including ecosystems) are closely linked and interdependent. The approach mobilizes multiple sectors, disciplines, and communities at varying levels of society to work together to foster well-being and tackle threats to health and ecosystems, while addressing the collective need for healthy food, water, energy, and air, taking action on climate change and contributing to sustainable development’ [9].

The *Kunming-Montreal Global Biodiversity Framework* is remarkable in affirming [where they are recognised] the ‘…rights of nature and rights of Mother Earth, [and are] an integral part of its successful implementation’ (p. 5), and even calls for a ‘One Health Approach’ (Section C(r)).

We therefore imagine Ecogenomics, as part of an aspirational *Ecological Genome Project* (inspired by the ambitious global endeavour of *The Human Genome Project*), to connect an *eco*logy built around the genomic sequencing of the world around us, to human *genomics*. We see such a Project as building on the significance of genes to cultures with natural history. The Project expands human ecology into a grand vision of our ‘home’ (from the Greek *oikos*)—the biosphere of Planet Earth—to connect the molecular and exposome study of human and non-human life, situated in shared environments and communities. These relationships affect us throughout our lives and are inheritable. The Project therefore started with the concept of exposomics and the scientific measurement of environmental exposures [10], but now is an opportunity for exploring further the ecological dimensions of health.

Ecogenomics concerns three areas.

First, genomics has been used as an approach to develop biotechnological opportunities (often from, or by modifying, ecoservices, e.g., modified compounds, and gene-edited crops) to achieve the Sustainable Development Goals (SDGs).^3^ COP15 emphasised that continued loss of biodiversity is linked to the social and environmental determinants of health (particularly SDG13 *Climate Action*, SDG14 *Life Below Water,* and SDG15 *Life on Land*) and requires strengthening the Nagoya principle of ‘benefit sharing’. How, then, does human genomics relate to the environmental SDGs and to biodiversity as implied by the ecological lens of the One Health approach?

Part of the answer comes in the second area: Ecogenomics recognises the ways the human genome is embedded in ecosystems and influenced by diverse environmental factors. It is the molecular study of the environmental influences on an organism’s genome, including the impacts of ambient agents on heritable variations (e.g. exogenous mutagens), or changes in the personal microbiome [11].^4^ Patterns of molecular, genetic, and epigenetic change must also be studied in ways that account for communities’ complex social histories, exposures to stress, and access to the basic resources and opportunities that promote community health, but that are *also* influenced by social ecology. (We know that sociality *with* animals, and *in* nature, impacts on our health. The COVID-19 pandemic illustrated how both were part of the narrative—e.g., contact with bats, lock downs with companion animals/without access to nature, and the ‘social lives’ of microorganisms [12].)

Thirdly, is the understanding that the environment is dynamic: it connects us to nature, sometimes in interdependent ways; it is a space we share with other biotic communities; and it signifies a natural history in which genomic similarities between species, in many respects, are more than the differences. This is an ethical, legal, and social investigation of our relationships with other species [8].

The common thread of Ecogenomics, therefore, is that human life on planet Earth relies on the diversity of other species. The visionary Ecological Genome Project is a global initiative to inspire the study of human well-being as a connection to non-human animals, and the plants and microbes around us, also recognising the importance of biodiversity, conservation, and ecology. Understanding these connections, dependencies, and interactions between the organisms that live here, and with which we share space and resources, reveals the importance of the ecological systems that sustain all of us. Their study is only possible as integrated multi-omics. In this respect, the approaches to genomics, including human epigenetics and the individual human microbiome, have begun to explore environmental DNA (e-DNA) and comparative genomic diversity of non-human species. But to be successful requires further integration of ‘eco’ sciences. Our present understanding may be limited by anthropocentric outcome measures. Expanding our vision includes taking research in unusual directions to explore radical solutions to find out who and what is interacting across environments, including species genomic variation and its relevance to resilience and susceptibility across the natural and social worlds.

Ecogenomics is the recognition of the situatedness of human beings and our relationships with other species and planetary health more widely. Our vision is one in which the human health/disease risk/phenotype paradigm is compatible with the international turn to ethical environmentalism.

## HUGO and Biodiversity Post-2020

In this perspective, HUGO CELS emphasises that the *Kunming-Montreal Global Biodiversity Framework* targets have relevance to genomic research institutions, directly or indirectly, involved in environmental research: as users of ecoservices; being responsible for reducing negative impacts on biodiversity; being producers of benefits with respect to the environmental determinants of health; and meeting biosafety measures with respect to such benefits. There is also a responsibility for all scientists to adapt genomics to sustainable futures. With respect to the SDGs, genomic scientists have a role in stabilising the ecological determinants of health, which requires interdisciplinary research, as well as cultural and social responsiveness, and responding to international governance challenges.

HUGO CELS purpose is to bring about cultural change within the scientific and clinical communities. Our vision, therefore, involves supporting many intellectual trajectories to achieve the *Kunming-Montreal Framework’s* Global Biodiversity Targets. For HUGO, this has been principally achieved by promoting *the public good*, advocating for *benefit sharing*, and exploring global governance. In its pioneer statement made in 2000, the HUGO Ethics Committee recommended that all humanity share in, and have access to, the benefits of genomic research [13]. Stated in clear and actionable terms, HUGO’s statement called for dedicating a percentage of commercial profit to public healthcare infrastructure and humanitarian efforts. Moreover, the statement emphasised that benefit sharing could not be achieved without the prior discussion with groups or communities who were impacted by the establishment and development of genetic resources. In the intervening decades, community engagement and indigenous data sovereignty have become more central to ethical research and data practices in ecology and genomics.^5^[14] In 2019, HUGO CELS (as it had by then become), reaffirmed the right of every individual to share in the benefits of scientific progress and its technological applications, as an expression of genomic solidarity. Solidarity was a prerequisite for an ethical open commons in which data and resources were shared.^6^ Reducing health inequalities among populations required promoting egalitarian access to the benefits of scientific progress, along with conditions for ethical access and use of genetic data [15].

HUGO’s response to the unfurling events of the COVID-19 pandemic included a call to effectively integrate environmental factors in public health genomics [16]. Two years on from the COVID Statement, the magnitude of the pandemic should be a wake-up call to operationalise all-of-knowledge responses to future zoonotic spillover events. The pandemic has also highlighted the connections between humans and animals in terms of resistance, resilience, and susceptibility: COVID-19 was both a zoonoses at the point at which it first infected a human community, and a reverse or zooanthroponosis as it infected non-human animals around the world. In this respect, there has been a growth in One Health approaches, defined by spatial, temporal, and organisational scales related to public health, that have *interspecific* benefits and centres environmental factors in studies and decision-making [8]. One Health is related to the concepts of ‘One Medicine’ and ‘Planetary Health’.^7^ With respect to the impact of the COVID-19 pandemic, these concepts can be combined to benefit non-human animals and humanity. For example, collectively harnessing the vast genetic data collected on non-human animals in the wild and as veterinary patients, can be integrated in all aspects of pandemic response planning, including building an innovative infrastructure for deep prevention of future public health risks; ‘green’ biobanks containing human, animal, and microbiological sequences; and strategies for environmental protection [19].

HUGO CELS also aims to foster scientific exchange in genomics between diverse academic and professional communities. It is therefore necessary for HUGO CELS to emphasise its conviction that Ecogenomics requires the mainstreaming of the One Health approach that includes the involvement of genomic scientists in interdisciplinary teams, working towards an integrated understanding of ethical environmentalism.

## A HUGO vision for Ecogenomics

Genomics will be fundamental to the global responses to the challenges of the Anthropocene: the unit of geologic time used to describe the influence of human activity on the planet's climate and ecosystems [20]. As a result of accelerating environmental change and its impact on the global burden of disease, there is a will to expand theories of environmentalism, developed in areas such as conservation, ecology, and veterinary public health, into clinical and epidemiological-based public health disciplines. To do so, we consider the concept of One Health as a framework for understanding planet Earth and all its life and matter that comprise the complex whole system—the Gaia 2.0 [21]. One Health is a concept fundamental to envisioning an Ecological Genome Project. Using a One Health paradigm, genomic sciences can contribute to the clinical and social responses to the environmental, economic, legal, and social determinants of health. In this regard, it is of critical importance that we build into that a mutual understanding of the ecological determinants of health that impact on all life [22]. A further step—justified under the One Health paradigm—will be progress in sharing the benefits of these technologies with non-human nature through innovations in these fields. In the light of the *Kunming-Montreal Global Biodiversity Framework*, integration should include the exploration of genetic diversity within populations of wild and domesticated species in terms of safeguarding their adaptive potential to be healthy, as well as being indicators and sentinels of imperilled environments. But ongoing adaptation of this paradigm already reveals tensions in public health [8]: the latter requires manipulating our surroundings through direct (engineering and design, rewilding, and environmental law) and indirect (environmental taxes and nudges) means, but rarely acknowledges the impacts these have on environmental sustainability and the recondite value of nature.

Our approach goes beyond the traditional combination of ecology and molecular biology, to create a holistic picture of the biotic and abiotic environment. Connecting human population health to environmental population data (such as studying the built environment and land use) is an established matter of study for cultural geography, conservation, landscape ecology, and social–ecological system studies. To the extent that the environmental determinants of health index our indelible place on and in the landscape, these areas identify the continual exchanges between communities with fauna and flora, across ecosystems. There is a longstanding assumption that human society benefits from cooperation in these systems, but that can only be achieved through understanding ethical factors affecting communities ‘in a place’. Studies of complex population characteristics, with feedbacks among various natural and social systems, show how cultural norms form [23]. These norms are strengthened by dialectic conditions for defining and transmitting our interests and values to others, to both those present and future generations, with respect to our prospects for healthy living and healthy environments.

The adaptation of clinical genetics to account for ecological genomics raises significant questions. It extends practices further into interspecies interactions that are outside of frames for the Ethical, Legal and Social Implications (ELSI) at the genesis of The Human Genome Project, where pertinent clinical and ethico-legal factors came to the fore. Since then, ELSI has become a lens for the social, historical, legal, and political determinants of health, and has developed a framework for environmental justice [24]. For Ecogenomics to contribute to solving complex societal problems, collaboration between scientific and non-scientific experts is crucial, but the conceptual function of environmentalism to aid this is unclear [25]. How do the ecological determinants of health—the processes in nature that impact the health and well-being of all species—integrate with this evermore complex genomic picture?

For example, the COVID-19 pandemic is a complex, all-of-society event: genomics concerned sequencing the SARS-CoV-2 virus and its variants, and exploring the environmental conditions that caused it to emerge and its evolutionary drivers. The human haplotype/pangenomic understanding focussed on susceptibility and vulnerability (and factors of moderate, severe, and critical illness), and contributed to the socio-economic drivers of infection, morbidity and mortality, and recovery [26]. Public health genetics studied natural reservoirs and hosts (but are yet to find the first reservoir), and the impact of infections in animals as mutation amplification risks, rather than welfare issues. A justification for the Ecological Genome Project, therefore, is to enhance and expand available responses to future pandemics which include intraspecific interest, such as animal welfare in general and opportunities like animal vaccination. It involves looking carefully at the ethics of trade, exploitative land use, and unsustainable use of ecoservices. In that respect, there is a vast amount of sequence and sample data from the COVID-19 pandemic, so this Project is a renewed awareness of the importance of biobanks of non-human biological specimens and ecological samples. We can use these further to generate data about the health of the biosphere; to create opportunities for socio-economic sustainability, e.g., in food access, and plant adaptability to climate and ecological changes; and to harness novel products.

There are a number of initiatives engaged in genomic sequencing, such as the PREDICT initiative led by the *EcoHealth Alliance*,^8^ and the *Global Virome Project* which is ‘a pathway to improve capacity to detect, diagnose, and discover viruses that potentially pose threats to human populations, particularly in low-income and middle-income countries’ [27]. *The Earth Biogenome Project* is a consortium that aims to sequence ‘Life for the Future of Life’ based on the principles of the Human Genome Project [28]. With the sequences of every genome of every eukaryote, we will better understand the impact of climate change on biodiversity, develop knowledge-based conservation efforts for endangered species and ecosystems, and better protect and enhance ecosystem services.

These genomic approaches raise ethical issues, which show there are not always prosaic synergies between ecologies and conservation, and with public and clinical health. In these respects, there will also be regional and international cultural, historical, social, and structural obstacles and dynamics to navigate. The Ecological Genome Project blueprint, discussed below, will require norms for access and use of biobanks and data, descriptions of qualified researchers (i.e., with a much wider scope of interest, there will be unconventional access requests, as well as the dangers of open access to pathogenic sequences) [29], and principles that promote diverse interests in clinical and veterinary medicine, as well as conservation and ecology. We envisage modified training in collecting and using the specimens and interpreting environmental-genomic exposure histories. Guidelines may need to be revised, forcing a critical review of existing protocols that could strengthen international sharing of samples and data. Cross-species analytical tools within this biological space will need development. It is imperative, therefore, that the challenges of implementing this vision are debated, eventually to provide a roadmap to engage governments and communities.^9^

## Conclusion

HUGO CELS proposes the exploration of Ecogenomics in the ‘spirit of One Health’—using its opportunities to break down silos, integrate environmental genomics across policy, and encourage the involvement of genomic scientists in regional and international collaboration. We propose a vision for the Ecological Genome Project: to discover the environmental/ecosystem, economic, ethical, legal, and social significance of all genomes in the ecological and environmental contexts. None of these areas should be necessarily prioritised above the others, so matters of hierarchies of expertise must be addressed. This applies not just to ‘scientific team’ dynamics, but also the social zeitgeists. In that respect, any resulting bioeconomy that emerges from an ecological lens—just like those that appeared subsequent to the HGP [30]—has the potential to bring innovative solutions but also has the potential to increase social inequalities for communities, and could increase avenues for private capture of significant biodiversity assets rather than their distribution as public goods.

Just as ELSI became an expanded view of ‘Gen-ethics’ to include the social histories and experiences of genetic sequencing, we believe that a ‘human-focused’ approach can and must move beyond anthropocentrism [31]. This aspirational effort—inspired by the emergent ELSI movement that became far more that just *one* ‘project’—will account for the local, regional, and global; and the cultural and natural differences across all determinants of health. It will draw upon the wide sources available in environmental humanities, bioethics, economics, and social sciences, to contribute to cultural and political responses to a crisis in humanity’s relationships with their surroundings. To move this blueprint forward, HUGO CELS suggests positioning Ecogenomics as:

1. A conceptual challenge to take account of our future through the ecological determinants of health, and to frame and respond to the environmental harms that many communities face now and in the future.
2. An opportunity for genomic sciences to integrate into interdisciplinary research, including identifying and responding to the socio-environmental and ecological drivers of health.
3. An exploration of the governance challenges emanating from the *Kunming-Montreal Global Biodiversity Framework* and international law.

There are opportunities for unique initiatives, especially in providing an ethical framework for implementing genomics and understanding results of ecogenomic research, with respect to:

- Utilisation of interspecific sequences, to develop shared genomic resources (an expanded expectation to use human and non-human sequences stored during the pandemic, as the bases for environmental initiatives as well as clinical ones).
- Genomics as a contribution to conservation, and protection and utilisation of bioindicator species.
- Using genomics to study the impacts of pollution and as an opportunity for bioremediation of climate change.
- Understanding ecosystem health through a complex conceptualisation of economics, ethics, law, and society.
- Protecting while studying natural resources, through the functions of ethical biobanks and the open commons, where normative principles support innovative use of new scientific knowledge, but also requires obligations to use it ethically [32].

In 2002, John Sulston and Georgina Ferry critically wrote that HUGO was (historically) ‘interested primarily in medical genetics rather than wider biological importance of genomes [33]’. Today, we recognise that the human genome cannot be seen in isolation from the environmental determinants of health and the genomic implications of non-human life that surrounds and is part of 'us'. This Ecological Genome Project is an aspirational opportunity to enhance HUGO’s mandate to include ecological genomics.

## Abbreviations

CBD: United Nations Convention on Biological Diversity
COP15: Conference of the Parties (United Nations Biodiversity Conference held in Montreal, Canada, in 2022)
ELSI: Ethical, Legal and Social Implications (of the Human Genome Project)
HUGO: Human Genome Organisation
HUGO CELS: HUGO Committee on Ethics, Law and Society
SDGs: Sustainable Development Goals

## Footnotes

1 CBD/COP/15/L.25; 18 December 2022.

2 However, we acknowledge that the CDB and Nagoya Protocol will require adaptations, perhaps through specific regulations based in international law, to provide for faster biological samples sharing during global emergencies such as emerging pandemics.

3 Target 13 of Global Biodiversity Framework requires that parties: ‘Take effective legal, policy, administrative and capacity-building measures at all levels, as appropriate, to ensure the fair and equitable sharing of benefits that arise from the utilization of genetic resources…’.

4 See the Human Microbiome Project: http://commonfund.nih.gov/hmp/

5 This supports the Nagoya Protocol requiring engagement with indigenous and local communities, with an understanding of and respect for their ‘customary laws, and their free, prior, and informed consent’. These broad measures have since been enhanced (and criticised in their limitations) by the Global Indigenous Data Alliance, who advocate for the CARE Principles for Indigenous Data Governance [Collective benefit, Authority to control, Responsibility, and Ethics]: https://www.gida-global.org/.

6 While there are concerns over accidental escape, virus discovery and sequencing might also be misused as a result of programmes like USAID’s DEEP VZN and the Global Virome Project, which currently seek to identify pandemic-capable viruses through laboratory characterisation and share them in a list rank-ordered by threat level.

7 These are related concepts to One Health. One Medicine is anchored in veterinary medicine and veterinary public health, and uniquely a lens for ‘…development, international health, aid and post-colonial reconstruction’ [17]. Planetary Health is the ‘health of human civilisation and the state of the natural systems on which it depends’ [18].

8 https://www.ecohealthalliance.org/

9 See Acknowledgements section about HUGO’s plan of work in this respect.
